# Introduction of ultrasound-guided axillary vein approach for central venous catheterization in severely injured trauma patients: characteristics and concerns

**DOI:** 10.3389/fmed.2025.1603778

**Published:** 2025-08-18

**Authors:** Ruonan Gu, Shanxiang Xu, Shouyin Jiang, Xiao Lu, Haizhen Wang, Xiaogang Zhao

**Affiliations:** ^1^Department of Emergency Medicine, Haiyan People’s Hospital, Haiyan, Zhejiang, China; ^2^Zhejiang Key Laboratory of Trauma, Burn, and Medical Rescue, Hangzhou, China; ^3^Zhejiang Province Clinical Research Center for Emergency and Critical Care Medicine, Hangzhou, China; ^4^Research Institute of Emergency Medicine, Zhejiang University, Hangzhou, China; ^5^Department of Emergency Medicine, Second Affiliated Hospital, Zhejiang University School of Medicine, Hangzhou, China

**Keywords:** axillary vein, central venous catheterization, venous thromboembolism, multiple trauma, ultrasound

## Abstract

**Background:**

The ultrasound-guided axillary vein approach for central venous catheterization (UAVC) demonstrates high success rates and low complications; however, its utilization in trauma care settings remains limited. This study aimed to characterize UAVC practices in a trauma intensive care unit (TICU) at a tertiary teaching hospital, specifically investigating optimal catheter positioning, procedure-related complications, and risk factors associated with catheter inaccurate placement and venous thromboembolism (VTE) development.

**Methods:**

A retrospective analysis was performed on trauma patients who underwent UAVC between October 2021 and April 2023. This analysis was based on electronic medical records. Details of patients, procedures, and instances of catheter misplacement were carefully documented. The immediate complications after UAVC, including pneumothorax, hemothorax, hematoma, arteriovenous fistula, arterial dissection, and skin infection, were recorded. Moreover, late-onset complications such as VTE and catheter-related bloodstream infections (CRBSI) were also noted. Logistic regression was utilized to determine the independent risk factors for non-optimal catheter tip placement and VTE.

**Results:**

A total of 132 UAVC cases were analyzed, with 113 (85.6%) performed by resident physicians and no immediate complications observed. The VTE incidence was 27.3%, particularly higher in elderly patients (≥ 65 years, 43.4%), and fever during TICU stay was noted in 55.3% of cases. Catheter-related infections occurred at a rate of 3.38 per 1,000 catheter days, with eight cases (6.06%) of catheter misplacement. Accurate placement was achieved in 29.8% of 121 patients, predominantly on the right side (40.4%). Factors influencing inaccurate placement included patient age [odds ratios (OR) 1.06, 95% confidence interval (CI) 1.02–1.10], obesity (OR 9.31, 95% CI 2.58–33.56), and left-side placement (OR 133.04, 95% CI 21.66–817.29), while patient age (>54 years), fever, and ventilation duration (>6.6 days) were associated with VTE development.

**Conclusion:**

In severely injured trauma patients, UAVC is associated with a high incidence of VTE and a low rate of optimal catheter tip positioning. Our findings underscore the necessity of standardized protocols to refine catheter tip placement and warrant further investigation through randomized controlled trials.

## 1 Introduction

Central venous catheterization (CVC) is essential for the early treatment of critically injured patients in the intensive care unit (ICU). There are over 5 million central venous catheters inserted annually in the United States (1). Despite their frequent use, concerns have been raised about associated complications such as pneumothorax, hemothorax, hematoma, pseudoaneurysm, infection, and more importantly, thrombosis (2). While not uncommon, these complications can be life-threatening (3–5). Studies indicate that over 15% of CVCs result in complications, with mechanical issues occurring in 5–19% of patients, infections in 5–26%, and thrombosis in 2–26% (1). Notably, technical difficulties during CVC insertion-such as multiple attempts-are strongly correlated with increased complication risks (6, 7), careful selection of the anatomic site and procedure optimization may help to minimize the puncture complications.

There are traditionally several anatomic sites available for CVC insertion, including the internal jugular vein, subclavian vein, and femoral vein. However, carrying out a uniform approach is often impractical due to differing protocols and patient variability. Furthermore, although ultrasound-guided catheter insertion has gained enough popularity, surveys have reported high proportions of the subjective opinion of unnecessary or not immediately available for ultrasound technique even in developed countries (8, 9). The landmark technique for catheter insertion seems more prevalent in low- and middle-income countries which has been associated with increased risk of serious complications like tension pneumothorax and hemothorax (10). The internal jugular vein is a frequent alternative but may be discommodious for trauma patients with severe head or cervical spine injuries. The use of the femoral vein route requires caution in the ICU due to a relatively higher risk of infection unless it is an emergency. Therefore, the search for the ideal CVC anatomic site for trauma patients continues.

The axillary vein, which becomes the subclavian vein at the lateral border of the first rib, may be a viable option. Its location outside the chest wall and away from the pleural cavity minimizes the risk of hemopneumothorax during the procedure (11). Ultrasound-guided puncture of the axillary vein can prevent major bleeding and arteriovenous fistulas, given its separation from the axillary artery (12). Moreover, if the axillary artery is inadvertently punctured, the bleeding is more manageable through manual compression.

The axillary vein approach for CVC was first reported by Nichalls in 1987 (13). Subsequent studies have compared the success rates of the first attempt, access time, and guidewire time between the axillary vein and other sites such as the internal jugular vein or subclavian vein for CVC (14). Currently, a growing trend is evident where an increasing number of trauma centers are attempting to employ the ultrasound-guided axillary vein approach for central venous catheterization (UAVC). To date, however, there is no literature reporting on the safety of UAVC in severely injured patients. Analyzing its application in this patient group is critical due to the lack of information on procedural details, optimal depth, and potential thrombotic complications, aside from its advantages, especially given the unique challenges of CVC placement in trauma patients. Since October 2021, our trauma intensive care unit (TICU) has implemented UAVC for critically ill trauma patients requiring precise volume and hemodynamic monitoring. This study aims to describe the characteristics of UAVC in our TICU, including optimal positioning and complications as well as related contributing factors. This data could provide a valuable reference for UAVC practice in trauma patients.

## 2 Methods

### 2.1 Study design

This retrospective observational study was conducted at the TICU in a tertiary referral hospital in China. The unit, affiliated to the National Trauma Regional Medical Center, has 10 approved beds, and treats over one thousand severe trauma patients each year. It operates with 1 fellow physician, 3–4 attending doctors, 12–20 resident doctors (mostly rotating), and 10 nurses at regular weekday (including weekends and holidays). We initiated UAVC performed by attending doctors in October 2021 and since then trained rotating residents to perform this procedure. The study received approval from the Human Research Ethics Committee of the Second Affiliated Hospital of Zhejiang University School of Medicine (approval no. 2023–0430) and was registered at ClinicalTrials.gov (NCT05896735). The work was reported in line with the STROCSS guidelines (15). Informed consent was waived due to the study’s retrospective nature and the absence of any personally identifiable information in the data collected.

### 2.2 Study population

Patients who were admitted to the TICU between October 2021 and April 2023 were eligible for study. The study included trauma patients who were between 0 and 100 years old and had undergone ultrasound-guided axillary venous catheterization. Patients without axillary venous catheterization records were excluded. Patients without imaging confirmation of catheter tip position were excluded for further analysis of associated factors. Guided by the principles of sample size estimation for logistic regression models, our primary outcomes of interest were inaccurate catheter tip placement and venous thromboembolism (VTE). The final sample size was determined as 132 patients, accounting for an estimated suboptimal catheter positioning rate of 60–70% and a VTE incidence of approximately 25–30% in this high-risk cohort. For logistic regression models, a minimum of 10 events per predictor variable is recommended to ensure model robustness (16). Accordingly, a sample size of 110 patients was identified as the minimum requirement during study design to assess risk factors for both suboptimal UAVC tip positioning and VTE using logistic regression. Given the necessity for potential stratified analyses, the sample size was subsequently increased by 20%.

### 2.3 Technique of the procedure

**Figure 1:**
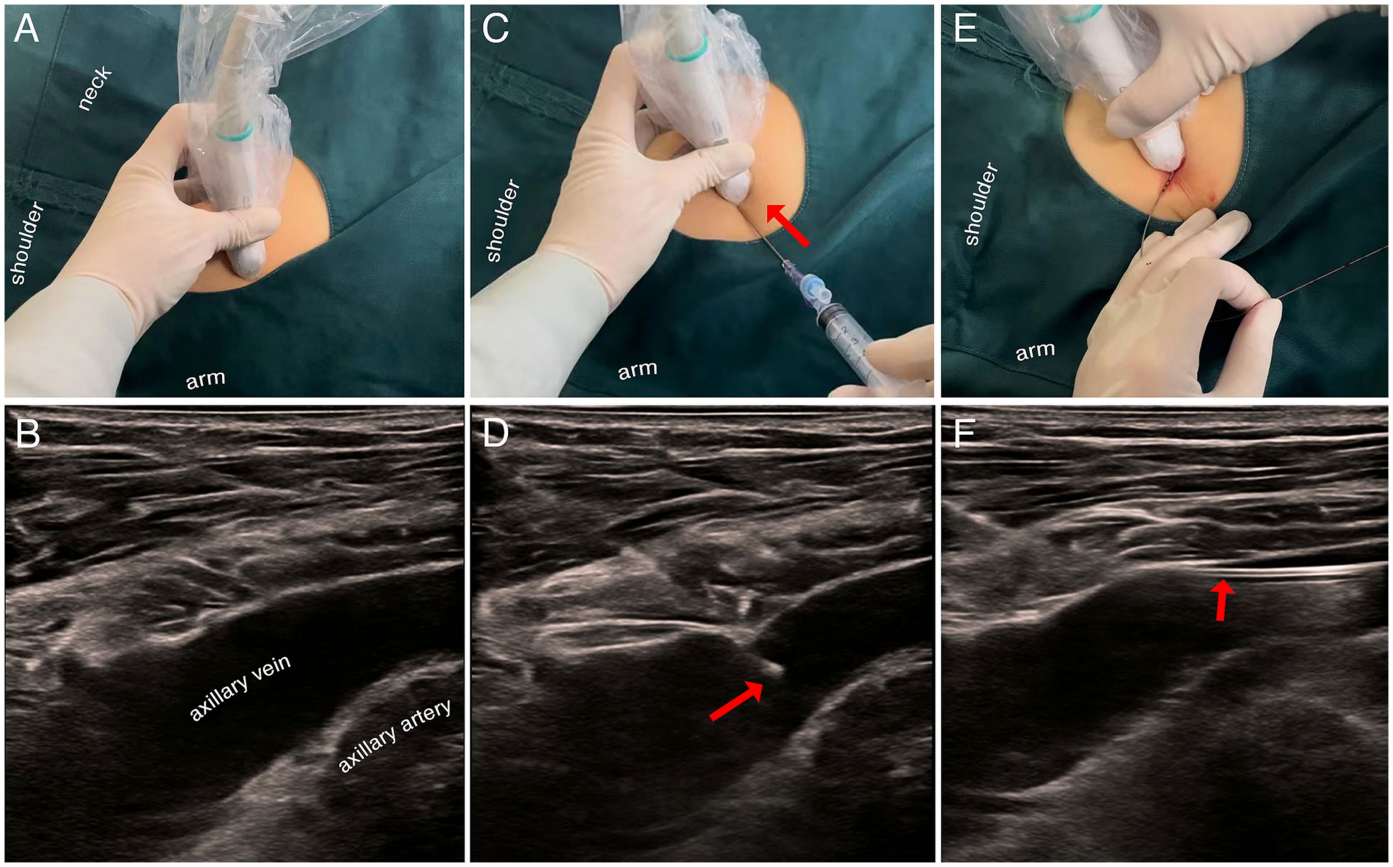
Representative steps for ultrasound-guided axillary vein approach for central venous catheterization (UAVC). **(A,B)** Long-axis (in-plane approach) ultrasound view of the axillary vein, with one hand resting against the shoulder to stabilize the view and reduce compression of the axillary vein. **(C,D)** The UAVC procedure is performed using an in-plane technique, clearly displaying the hyperechoic puncture needle within the vessel lumen. **(E,F)** Confirm the placement of the guidewire with ultrasound before inserting the catheter.

We adhered to the standard protocols for CVC using bedside ultrasound guidance for axillary vein access (Figure 1) (17). The procedure mainly includes: (1) obtaining informed consent from the patient’s relatives; (2) Ensuring cooperation from conscious patients or administering appropriate sedation and analgesia to those comatose; (3) Positioning the patient supine, with arms by their sides or slightly abducted; (4) Utilizing ultrasound to distinguish the axillary vein from the artery, evaluate catheterization difficulty, and opt for jugular or subclavian vein access if necessary due to venous collapse or excessive depth of veins; (5) Disinfecting the skin with chlorhexidine, covering with a sterile drape, and placing the ultrasound probe and cable in a sterile sheath; (6) Using ultrasound guidance to confirm the insertion site, applying lidocaine for local anesthesia, and inserting the needle under negative pressure, ensuring the insertion point is 0.5–1.0 cm from the ultrasound probe’s skin contact point in the axillary vein’s long-axis view; (7) Inserting the guidewire upon obtaining dark red blood, verifying its position within the vein with ultrasound; (8) Cannulating using the Seldinger technique and flushing all ports with normal saline to ensure patency; (9) Confirming the catheter tip’s position with a bedside chest X-ray or a scheduled CT scan.

### 2.4 Data collection

We extracted the following data from our electronic medical records (EMRs): procedure date, patient age, gender, height, weight, body mass index (BMI), existing comorbidities, Acute Physiology and Chronic Health Evaluation II (APACHE II) score, Injury Severity Score (ISS), Caprini Risk Score (CRS), injury diagnosis, records of mechanical ventilation and continuous renal replacement therapy (CRRT), pre-cannulation blood routine examination and coagulation function tests, immediate complications related to the insertion, thrombotic events, duration of catheter indwelling, and occurrences of fever. Additionally, we gathered information on the procedural operator’s level through the qualification system. For each case, we manually reviewed the imaging history post-UAVC.

### 2.5 Definition

Fever was defined as a body temperature exceeding 38.3 degrees Celsius, according to the consensus of the American College of Critical Care Medicine and Infectious Diseases Society of America (18). Immediate complications following UAVC within 24 h included pneumothorax, hemothorax, hematoma, arteriovenous fistula, arterial dissection, and skin infection. These were identified through imaging findings and medical records. Late complications were VTE and catheter-related bloodstream infections (CRBSI) during the stay in the TICU. VTE, including deep vein thrombosis (DVT) and acute pulmonary embolism (APE), was diagnosed through lower limb venous ultrasound or CT pulmonary angiography. CRBSI was defined as a primary bloodstream infection occurring within 48 h after central venous catheter (CVC) placement, unrelated to any other infection sites (19). The rate of CRBSI was calculated per 1,000 catheter-days (20). Elderly patients were defined as those with an age of 65 years or older. The optimal position for a CVC was defined as the catheter tip being within 2.0 cm of the tracheal carina level, with deviations from this range defined as positive or negative (namely non-ideal catheter tip). This positioning is crucial for accurate central venous pressure (CVP) measurement or pulse indicator continuous cardiac output (PICCO) monitoring (21–24). Patient discharge outcomes included death, self-discharge due to deteriorating conditions, and discharge with improvement, either to a general ward or a local hospital. Prolonged prothrombin time (PT) was defined as ≥15.0 s (25), and hypofibrinogenemia as fibrinogen levels < 2.0 g/L (26). A platelet counts lower than 100 × 10^9^/L was considered thrombocytopenia (27).

### 2.6 Statistical analysis

Categorical variables are represented as counts and percentages, while continuous variables are shown as medians with interquartile ranges. To compare categorical variables between groups, we employed the Chi-square test or Fisher’s exact test for expected counts below five. The Mann–Whitney *U* test was used for continuous variable comparisons. We used logistic regression analysis to identify independent risk factors for inaccurate CVC tip placement and the occurrence of VTE during TICU stays. Variables with a *p*-value less than 0.1 in univariate analyses were included in the multivariate logistic regression model. We developed the final model using the forward stepwise LR method, presenting results as odds ratios (ORs) with 95% confidence intervals (CIs). Analysis was performed using IBM SPSS Statistics (version 29.0), with a *p*-value of less than 0.05 considered significant. Furthermore, receiver operating characteristic (ROC) curve analysis was performed to determine the optimal cut-off values of risk factors for VTE. ROC curves were constructed using MedCalc software (Version 19.0.4).

## 3 Results

### 3.1 Patients’ characteristics

**Figure 2:**
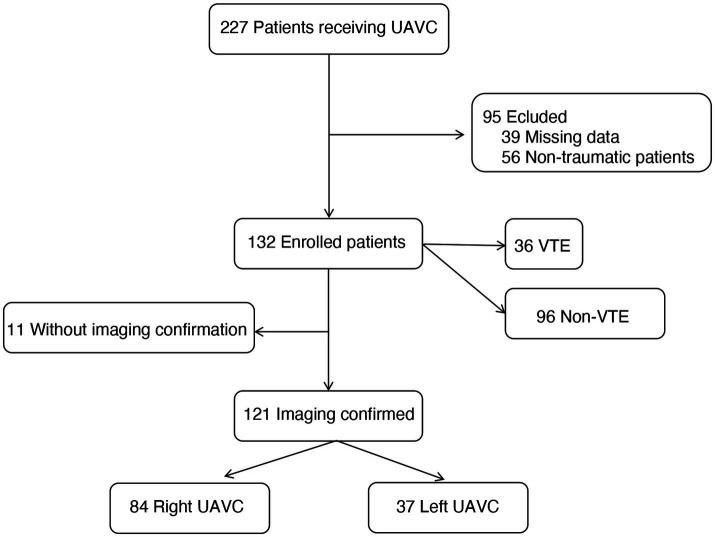
Flow diagram of study patients.

During the study period, 227 UAVCs were performed in the TICU. After excluding non-trauma patients and those with missing data, 132 trauma cases remained for primary analysis. Resident physicians performed 85.6% of the UAVC procedures, with 63.4% occurring during regular weekdays and 24.2% on weekends and holidays. Eleven cases were excluded from the secondary analysis due to the absence of imaging localization (Figure 2). The demographic data of the study participants are detailed in Table 1. Of the cannulations, 68.18% were performed in the right axillary vein. There were no significant differences in age, gender, BMI, APACHE II score, ISS score, coagulation status before cannulation, and use of ventilators or CRRT between the right and left side groups. Specifically, 20.5% of patients experienced thrombocytopenia, with 3.8% having platelet counts below 50 × 10^9^/L, the lowest being 21 × 10^9^/L. Furthermore, 10.6% of patients exhibited hypofibrinogenemia, with the lowest fibrinogen level recorded at 0.73 g/L. Prolonged PT was observed in 31.8% of patients, with the maximum PT extending to 29.5 s.

**Table 1:** Demographic characteristics of the studied patients.

Items	Overall	Right placement	Left placement	*P*-value
	(*n* = 132)	(*n* = 90)	(*n* = 42)	
Age, years	60 (49–70)	60 (50–70)	58 (47–71)	0.907
Age group				1.000
Adolescent (<18 years)	6 (4.5)	4 (4.4)	2 (4.8)	
Young and middle-aged	73 (55.3)	50 (55.6)	23 (54.8)	
Elderly (≥65 years)	53 (40.2)	36 (40.0)	17 (40.5)	
Male	97 (73.5)	66 (73.3)	31 (73.8)	0.954
Body mass index, kg/m^2^	22.86 (20.97–25.29)	22.86 (21.37–25.08)	22.68 (20.76–25.39)	0.321
APACHE II score	13 (9–20)	12 (9–19)	17 (8–25)	0.142
Caprini score	10 (8–12)	9 (8–11)	11 (8–13)	0.039
ISS	25 (17–30)	24 (17–29)	26 (19–31)	0.356
Coagulation before cannulation				
Platelet count, ×10^9^/L	164 (110–203)	169 (110–207)	141 (110–193)	0.254
Fibrinogen, g/L	3.33 (2.43–4.97)	3.10 (2.28–4.71)	4.1 (2.43–5.51)	0.083
Prothrombin time, s	14.1 (13.4–15.3)	14.1 (13.5–15.3)	14.1 (13.4–15.4)	0.971
Comorbidity				0.025
Circulatory system	27 (20.5)	20 (22.2)	7 (16.7)	
Respiratory system	3 (2.3)	3 (3.3)	0 (0)	
Nervous system	2 (1.5)	1 (1.1)	1 (2.4)	
Diabetes	4 (3.0)	1 (1.1)	3 (7.1)	
Multiple comorbidities	19 (14.4)	17 (18.9)	2 (6.3)	
Musculoskeletal system	4 (3.0)	4 (4.4)	0 (0)	

Continuous and categorical variables are presented as median (interquartile range) and number (%), respectively. *p*-values are based on Chi-square test or Fisher’s exact test for categorical variables and Mann–Whitney *U* test for continuous variables.

### 3.2 Catheterization results

**Figure 3:**
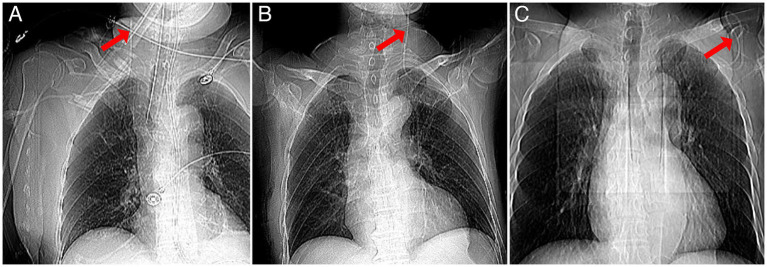
Representative images of catheter malposition in three trauma patients. **(A)** Misplacement of the catheter tip into the right internal jugular vein; **(B)** misplacement of the catheter tip into the left internal jugular vein; **(C)** subcutaneous catheter misplacement due to distortion.

Among the 121 catheters confirmed to be in position, only 29.8% were ideally placed. Chi-square analysis showed a higher incidence of non-ideal catheter tip positions on the left side compared to the right (59.5% vs. 94.6%, *p* < 0.001). Seventy-five percent of the catheters remained in place for less than 7 days, 22.0% for 7–14 days, and 3.0% for more than 14 days, with the longest indwelling time being 18 days. Eight catheter tips were mispositioned: seven in the jugular vein and one in the axillary vein, attributed to subcutaneous distortion (Figure 3).

### 3.3 Procedure-related complications

No immediate procedural complications were identified following UAVC procedures. Late complications included a 27.3% occurrence of VTE in patients. The DVT incidence stood at 25.8%, with rates broken down as follows: 0.0% in adolescents, 19.2% in young and middle-aged individuals, and 43.4% in the elderly. Pulmonary embolism occurred in 6.8% of patients. The development of VTE did not significantly differ between patients with left side UAVC (28.6%) and those with right side UAVC (26.7%). Fever during TICU stay was reported in 55.3% of patients, with more than half experiencing temperatures ranging from 38.3 to 39 °C. The CRBSI rate was 3.38 per 1,000 catheter-days.

### 3.4 Factors associated with non-ideal catheter tip position and VTE

**Figure 4:**
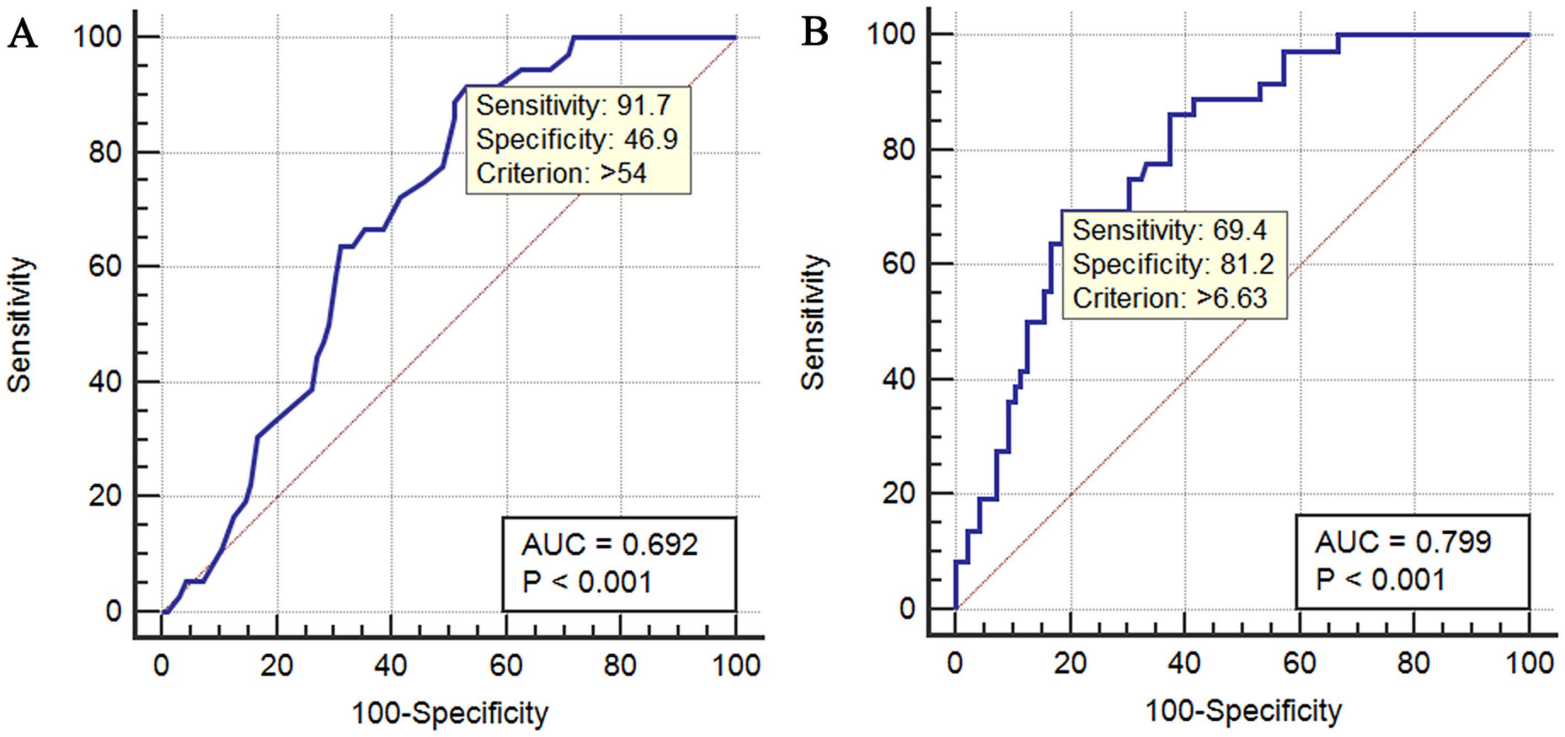
Receiver operating characteristic (ROC) curves for predicting venous thromboembolism (VTE) in trauma patients undergoing ultrasound-guided axillary vein catheterization (UAVC). Age **(A)** exhibited an AUC of 0.692 (*p* < 0.001) for VTE prediction. At the optimal cutoff of >54 years, the model demonstrated 91.7% sensitivity but 46.9% specificity, supporting utility for screening but with a risk of overdiagnosis. Duration of mechanical ventilation **(B)** showed superior performance (AUC 0.799, *p* < 0.001). At the optimal cutoff of >6.63 days, it achieved 69.4% sensitivity and 81.2% specificity, outperforming age in differentiating VTE from non-VTE cases.

Univariate logistic regression analysis identified several factors for multivariate analysis: age (OR 1.021, 95% CI 0.999–1.043, *p* = 0.059), being overweight or obese (OR 2.028, 95% CI 0.954–4.312, *p* = 0.066), and left side placement (OR 32.515, 95% CI 10.130–104.367, *p* < 0.001). Night-time catheter placement (OR 0.453, 95% CI 0.206–0.996, *p* = 0.049) and procedures performed by resident doctors (OR 2.825, 95% CI 0.870–9.166, *p* = 0.084) were also included. The multivariate logistic regression model revealed that age, being overweight or obese, and left side placement were independent factors for non-ideal catheter tip positioning (Table 2). Additionally, age, APACHE II score, Caprini score, PT, fever, duration of prophylactic anticoagulation, catheter indwelling time, CRRT, and ventilation duration were deemed suitable for multivariate analysis for predicting VTE, each with a single *p*-value < 0.1 in the univariate analysis. The multivariate logistic regression analysis indicated that age, fever, and ventilation duration were independent risk factors for the development of VTE (Table 3). ROC curve analysis further validated the predictive performance of age and ventilation duration for VTE in trauma patients undergoing ultrasound-guided axillary vein catheterization (UAVC) (Table 4 and Figure 4). Age yielded an area under the curve (AUC) of 0.692 (95% CI: 0.606–0.770, *p* < 0.001). Using a cutoff of >54 years, it demonstrated 91.67% sensitivity (minimizing missed VTE cases) but only 46.88% specificity (increasing false positives), indicating utility as a screening tool with a trade-off of overdiagnosis risk. Ventilation duration exhibited superior discriminative ability, with an AUC of 0.799 (95% CI: 0.721–0.864, *p* < 0.001). At a cutoff of >6.6 days, it achieved 69.44% sensitivity (moderate risk of missed cases) and 81.25% specificity (minimizing false positives), thereby outperforming age in distinguishing VTE from non-VTE cases.

**Table 2:** Risk factors analysis for non-ideal catheter tip position in trauma patients undergoing ultrasound-guided axillary vein catheterization (UAVC): multivariate logistic regression analysis (121 cases, Forward, *α* = 0.05).

Factors	*B*	SE	Wald	OR (95%CI)	*p*-value
Patient age	0.058	0.021	8.080	1.06 (1.02–1.10)	0.004
Overweight or obese	2.231	0.654	11.616	9.31 (2.58–33.56)	<0.001
Left position	4.891	0.926	27.882	133.04 (21.66–817.29)	<0.001
Night indwelling	−0.852	0.589	2.098	0.43 (0.14–1.35)	0.148
Resident doctor operated indwelling	0.386	0.726	0.282	1.47 (0.35–6.10)	0.595
Constant	−10.096	2.295	19.355	0.000(−)	<0.001

OR, odds ratios; CI, confidence interval.

**Table 3:** Risk factors analysis for venous thromboembolism (VTE) in trauma patients undergoing ultrasound-guided axillary vein catheterization (UAVC): multivariate logistic regression analysis (132 cases, Forward, α = 0.05).

Factors	*B*	SE	Wald	OR (95%CI)	*p*-value
Patient age	0.061	0.017	12.657	1.06 (1.03–1.10)	<0.001
Fever	1.291	0.541	5.697	3.64 (1.26–10.50)	0.017
Ventilation time	0.151	0.04	14.153	1.16 (1.08–1.26)	<0.001
Constant	−6.477	1.295	25.025	0.002(−)	<0.001

OR, odds ratios; CI, confidence interval.

**Table 4:** Receiver operating characteristic (ROC) curve analysis of age and ventilation duration as predictors for venous thromboembolism (VTE) in trauma patients undergoing ultrasound-guided axillary vein catheterization (UAVC) (132 cases).

Variables	AUC (ROC)	95% CI	Cut-off	Sensitivity	Specificity
Age (years)	0.692	0.61–0.77	>54	91.7%	46.9%
Ventilation days	0.799	0.72–0.86	>6.6	69.4%	81.2%

UAVC, ultrasound-guided axillary vein approach for central venous catheterization; AUC (ROC), area under the receiver operating characteristic curve; CI, confidence interval.

### 3.5 Patient outcome

Among the 132 patients, the median length of stay in the TICU was 5 days (interquartile range, 1–9), with the median duration for mechanical ventilation being 4 days (interquartile range, 0–9). About 76.5% of the patients required ventilators, while 14.4% underwent continuous renal replacement therapy (CRRT). The in-hospital mortality rate stood at 6.1%. At discharge, 12.9% of the patients showed deterioration, whereas 78.8% were transferred to general wards or local hospitals.

## 4 Discussion

To the best of our knowledge, this represents the first report from a single institution setting documenting the utilization of UAVC in trauma patients. Our study demonstrated that UAVC performed in TICU is associated with unsatisfactory catheter tip positions and high incidence of late complications such as VTE. Factors associated with non-ideal catheter tip position included patient age, being overweight or obese, and left side placement. Patient age (>54 years), fever, and ventilation duration (>6.6 days) were independent risk factors for the development of VTE.

The proper placement of central venous catheters is a critical aspect of clinical care. It is also essential to be aware of potential complications and take steps to prevent them. Identifying an ideal site for CVC in trauma patients is challenging due to various complicating factors. For instance, trauma patients often undergo surgeries that can make the internal jugular and subclavian veins unsuitable for catheterization. Furthermore, the specific positioning required to accommodate certain injuries can make catheter placement difficult.

Literature indicates that subclavian venous catheterization has a lower risk of bloodstream infection and symptomatic deep vein thrombosis than internal jugular and femoral venous catheterizations (28). Nonetheless, it has a higher risk of mechanical complications, such as pneumothorax. Incorrect puncture and catheterization techniques can lead to severe complications like hemopneumothorax, which may impair breathing and circulation. Moreover, the clavicle’s proximity makes hemostasis challenging if the subclavian artery is accidentally punctured.

Cannulation of the internal jugular vein may cause injuries, including posterior wall penetration or internal carotid artery damage, particularly in patients with hypovolemia. Inadequate coagulation can result in hematoma formation, potentially leading to tracheal compression, respiratory failure, or more seriously, cardiac arrest (3). Incorrect puncture methods or multiple attempts may increase the likelihood of complications such as dissection at the puncture site and cerebral embolism due to unilateral thrombosis. Using a femoral venous catheter can increase the risk of infection and may limit the use of certain intensive care monitoring techniques, such as central venous pressure and PICCO monitoring (28). As trauma patients often have complex conditions and unstable vital signs, minimizing the risk of additional harm is essential. Thus, the selection of the most suitable catheterization technique is critical for these individuals.

UAVC is an alternative. It does not require special patient positioning. The anatomical position of the axillary vein is beneficial as it is superficial, located outside the thoracic cavity, and well separated from the pleural dome (12). This reduces the risk of accidental pneumothorax and intercostal arterial injury compared to the subclavian vein access. In cases of axillary artery puncture, rapid compression can effectively control bleeding. Ultrasound-guided punctures allow for direct visualization, improving safety, success rates, and reducing the risk of mechanical complications from repeated or blind attempts (29–34). According to our data, 85.6% of ultrasound-guided axillary venous catheterizations were successfully performed by residents without complications, indicating that the procedure is straightforward and can be executed effectively by trained doctors.

The success of this procedure hinges on several factors, which include the following: Operators should have a thorough understanding of ultrasound operation before performing the puncture. For ultrasound evaluations, the longitudinal approach is preferred, as it has a higher one-attempt success rate (89.2% vs. 76.5%) than transverse approach by experienced operators (35). The positive results mentioned are reliant on specific puncture conditions.

During the puncture process, three critical considerations must be addressed. First, it is essential to control the application of force precisely. This encompasses both the pressure exerted by the ultrasound probe on the patient and the force used by the needle to penetrate the blood vessel wall. Lack of proper control can cause the axillary vein to collapse under the probe or result in the needle piercing through the posterior vessel wall. Second, the usually adopted needle insertion angle of 30–45 degrees is more suitable for patients who are thin or of moderate size. For obese patients, the axillary vein can be as deep as near 5 cm due to thicker layers of subcutaneous fat and muscles, which may require an increased insertion angle of up to 60 degrees. Positioning the needle bevel upwards also facilitates the correct direction of the guidewire for easier blood vessel penetration. Third, after the guidewire is inserted, ultrasound is utilized once more to ensure that the catheter is correctly placed within the axillary vein and directed toward the superior vena cava (17).

To support cardiorespiratory function, obtaining an accurate central venous pressure for monitoring is crucial; this is the same for administering individualized fluid therapy (36). Accurate placement of the catheter tip is key to this measurement. It should be positioned near the right atrium without entering it, ideally at the carina level, within 2 cm below this landmark. A shallow placement may yield a falsely high-pressure reading, whereas a deep placement can result in a falsely low reading. In our study, less than a third of patients had the catheter positioned ideally. The choice of catheter placement on the left or right side significantly affects accuracy. Our analysis showed that unsatisfactory positions were more common on the left, with 94.6% being too shallow. This may relate to the anatomical path to the superior vena cava. We recommend using the right axillary vein as the primary site to increase the chance of ideal positioning. We can also consider proximal axillary vein as needed, as studies have showed that it is associated with higher first puncture success rate and shorter cannulation time. It should be noted, however, that while patient age, obesity, and left-sided catheter placement were identified as factors associated with inaccurate catheter placement in our analysis, the absence of intraprocedural localization techniques likely exacerbated the influence of these variables.

The incidence of VTE in our patient group is comparable to the rates observed in trauma patients with central venous catheters in earlier studies (37–40). Our study identifies patient age and fever as independent risk factors for VTE, in line with previous research that highlights patient age, prolonged immobilization, obesity, and a history of thrombosis as associated factors (41, 42). This underscores the need for standardized management, incorporating both physical and pharmacological prophylaxis, to address the high incidence of VTE. Contact isolation has been strongly linked to VTE in trauma patients (43). The frequent occurrence of fever in ICU patients underscores the importance of focusing on infection prevention and control within the unit. The rates of catheter-related infections are consistent with historical data on central venous catheters (44, 45). Meanwhile, catheter indwelling duration is subject to several unpredictable factors. These include the patient’s recovery leading to a transfer, voluntary discharge due to a sudden health decline, death, medical condition changes during the hospital stay, bloodstream infection risk considerations, VTE, and proactive catheter replacement. As a result, the maximum safe duration for axillary venous catheterization has yet to be established.

Several challenges exist in UAVC. In patients with suboptimal puncture conditions, operators may resort to fluid replenishment to boost blood volume and use the Trendelenburg position to distend the vein. Skilled doctors may wait until the patient’s exhalation then insert the needle into the vein, when the vessel is temporarily dilated. However, if rehydration and the head-down position do not adequately enlarge the vein, and significant respiratory variation exists, successful catheterization becomes difficult. In such cases, delaying the procedure or selecting an alternative site may be advisable. Furthermore, the catheter tip misplacement into the internal jugular vein occurs occasionally, as shown by eight instances in 132 cases in this study. Therefore, performing an ultrasound after placing the guidewire is crucial to confirm its position and direction. Concurrent scanning of the internal jugular vein is advisable to detect and promptly correct any misplacement.

During UAVC, there exists a situation that despite the needle being in the vessel, inserting the guidewire can meet with resistance at around 10 cm depth. In such cases, we recommend holding the guidewire steady and withdrawing the needle, then using ultrasound to pinpoint the issue. If the guidewire has exited the vessel, re-insertion is necessary. If it is heading toward the axillary vein’s distal end or branches, partially retract it against the vessel wall under ultrasound guidance. Then, gently rotate and direct it toward the superior vena cava using its stiffness. Care must be taken to avoid pulling the guidewire too hastily or extensively, which might lead to its exit from the vessel and require re-puncture. These issues may arise from the needle bevel not facing upwards, an overly steep insertion angle, incorrect guidewire insertion technique, or insufficient vessel filling.

As highlighted by Annetta et al. (46), ultrasound-guided venous puncture in the infraclavicular region corresponds predominantly to axillary vein cannulation. Precise delineation of the anatomical boundaries between the axillary and subclavian veins, coupled with the employment of standardized nomenclature, is crucial for standardizing ultrasound-guided axillary vein catheterization in trauma patients and mitigating misinterpretation of complications-findings that underscore the necessity of focusing on UAVC technical optimization in the present study. Based on our results, we have developed key steps to optimize UAVC. Firstly, ensure good insertion conditions, adequate vascular filling, low respiratory variability, no local thrombosis, no vascular damage, no anatomical variations, and the distance from the insertion point to the vascular wall within 5 cm measured by ultrasound. Secondly, choose wisely, with priority given to the right axillary vein over the left for hemodynamic and volume support, and consider the proximal axillary vein as required. Thirdly, prepare for a sufficiently large disinfection area by thoroughly disinfecting the skin over the insertion site and adjacent areas where the internal jugular and subclavian veins are located to ensure sterility and clear visualization of the guidewire’s path. Fourthly, make adequate probe preparation by ensuring the probe is well-sealed with an ultrasonic coupling agent and a plastic sheath for a clear ultrasound image. Fifthly, hold the probe tightly with the hypothenar side of the palm resting on the skin to ensure a stable ultrasound image. Sixthly, apply appropriate force to ensure the probe does not flatten the blood vessels and the puncture needle does not penetrate the posterior wall of the vessels. Seventhly, adjust the probe angle appropriately as necessary to clearly visualize the blood vessels; generally, an angle between 30 and 45 degrees is recommended for shallow insertions, and a steeper angle like 60 degrees should be used when the distance from the insertion point to the vascular wall is long to avoid unnecessary penetration or damage. Eighthly, catheter tip confirmation must be done through bedside X-ray or a planned CT. Finally, complete the procedure quickly after advancing the catheter over the guidewire, and remember to flush all lumens to remove any residual blood.

## 5 Strengths and limitations

### 5.1 Strengths

The strength of this study lies in the fact that all data were obtained from electronic medical records, representing real and objective information. Although the sample size is small, this is currently the only study in the literature specifically reporting the application of this technology in hundreds of cases involving severe trauma patients, conducted in a large teaching hospital. It serves as a valuable summary that can provide a data reference for clinicians in other hospitals.

### 5.2 Limitations

Our study has limitations. The retrospective nature from a single center may bias the results. Single-center studies may not reflect outcomes from other hospitals accurately. With a sample size of 132 cases, the findings might not extend to a larger population. The retrospective design also raises concerns about missing or unrecorded data, potentially skewing the results. The absence of reported local complications might also be attributable to unrecorded events. The lack of real-time, intraprocedural catheter tip localization represents a notable limitation of our study. During the period from 2021 to 2023, our institution relied solely on post-insertion bedside imaging (X-ray or CT) to verify catheter tip placement, whereas contemporary techniques for intraprocedural guidance, such as electrocardiographic (ECG) monitoring or ultrasound-based methods-were not yet integrated into standard practice (47). This approach inherently introduces delays in confirming optimal tip positioning and precludes immediate corrective actions, which likely contributed to the suboptimal rate of correctly positioned catheters observed in our cohort (29.8%). Future investigations should prioritize the use of real-time localization modalities to enhance the accuracy of central venous catheter placement and minimize associated complications.

Notably, our study is subject to limitations regarding generalizability to broader populations, particularly in terms of age stratification. Given that our trauma center primarily serves patients aged ≥ 14 years, our cohort included only 6 adolescent cases (<18 years, 4.5%) with no children <14 years. Notable differences exist between adult and pediatric populations in axillary vein anatomy, physiological responses, and inherent complication risks, which may result in divergent outcomes related to ultrasound-guided axillary vein catheterization (UAVC). Accordingly, our findings regarding risk factors such as age and obesity cannot be directly extrapolated to pediatric populations, underscoring the necessity of dedicated investigations in pediatric trauma cohorts to establish age-specific clinical protocols. Furthermore, while our study focuses exclusively on trauma patients, UAVC may exhibit distinct risk profiles in other clinical contexts-including pediatric oncology and critical care settings (48). Specific protocols for UAVC in pediatric patients, including risk factor assessment, warrant further dedicated investigation.

Regarding VTE outcomes, catheter-related thrombosis is defined as a blood clot present in the vein that houses the catheter, or on the adjacent walls of the venous catheter after insertion. Due to the lack of evidence and data concerning upper limb thrombosis, this study cannot definitively establish a link between VTE and the use of indwelling deep venous catheters. Furthermore, it cannot precisely determine the actual rates of catheter related VTE. Moreover, given that the data collected during the COVID-19 pandemic were incorporated into this study, it is likely that the related infection prevention and control measures have exerted an impact on the research findings. Finally, our study did not conduct a direct comparison between UAVC and alternative CVC approaches regarding the proportion of procedures performed by resident physicians or differences in early and late complication rates. Consequently, definitive conclusions about the potential superiority of UAVC cannot be drawn from this investigation. However, this study represents pioneering work that synthesizes actionable improvement strategies derived from current data insights. These findings provide a robust clinical foundation for designing optimized protocols in future prospective comparative studies.

## 6 Conclusion

In summary, performing UAVC in severely injured trauma patients faces challenges such as a significant number of suboptimal catheter tip positions and a high incidence of VTE. Our findings highlight the need for standardized protocols to optimize catheter tip placement and mitigate VTE risks through early anticoagulation in elderly or ventilated patients. These adjustments could enhance safety in trauma ICU settings. Future studies should focus on assessing the incidence of VTE, comparing the efficacy of various puncture sites, and evaluating the potential of UAVC against alternative methods. Additionally, the effectiveness of the suggested catheterization optimization bundle requires validation through a randomized controlled trial.

## Data Availability

The raw data supporting the conclusions of this article will be made available by the authors, without undue reservation.
